# Supplementary material for: A Mesozoic clown beetle myrmecophile (Coleoptera: Histeridae)
Source: eLife. 2019 Apr 16;8:e44985. doi: 10.7554/eLife.44985 (PMC6467565; doi:10.7554/eLife.44985)
Supplement: Supplementary file 2. [file elife-44985-supp2.docx]

**Supplementary File 2A. Taxa sampled for phylogenetic analysis.**

| Subfamily | Tribe | Species |
| --- | --- | --- |
|  |  | **†***Promymister kistneri* |
| Histerinae | Histerini | *Hister unicolor* |
|  | Omalodini | *Omalodes grossus* |
|  | Platysomatini | *Platysoma punctigerum* |
|  | Hololeptini | *Hololepta vicina* |
|  | Exosternini | *Arbolister* sp. |
|  |  | *Baconia loricata* |
|  |  | *Coelocrara cohici* |
|  |  | *Coproxenus marshal* |
|  |  | *Cypturus aenescen* |
|  |  | *Epitoxasia* sp. |
|  |  | *Exosternus sp.* |
|  |  | *Exotoxus crypticus* |
|  |  | *Hypobletus subridens* |
|  |  | *Hypobletus taciturnus* |
|  |  | *Lactholister tricinctus* |
|  |  | *Megalocraerus rubricatus* |
|  |  | *Operclipygus foveiventr* |
|  |  | *Operclipygus lissipygus* |
|  |  | *Operclipygus sulcistrius* |
|  |  | *Paratropus wenzeli* |
|  |  | *Phelister rouzeti* |
|  |  | *Platybletes stirpium* |
|  |  | *Procoryphaeus wallace* |
|  |  | *Sabahister philippinensis* |
|  |  | *Sitalia severin* |
|  |  | *Xenosternus saprinopterus* |
|  |  | *Yarmister barberi* |
|  |  | *Yarmister emersoni* |
|  |  | *Yarmister MGSul* |
|  |  | *Yarmister* sp 1‒10 |
| Haeteriinae | Haeteriini | *Haeterius morsus* |
|  |  | *Haeterius ferrugineus* |
|  |  | *Homalopygus sp.* |
|  |  | *Thaumataerius sp.* |
|  | Synoditulini | *Synoditulus sp.* |
|  | Nymphistrini | *Nymphister monotonus* |

**Supplementary File 2B. Ages of haeteriine host ant genera inferred from fossil and molecular data.** Ages are millions of years. Palaeolocalities of fossils are given. Superscript numbers are citations containing data points and ages. We include the most recently-published molecular age estimates of crown-group taxa. Fossils have typically not been placed phylogenetically, so may be stem- or crown-groups; consequently, their ages exceed the molecularly-inferred crown-group age.

| **Ant taxon** | **Oldest fossil age^30,31^** | **Crown–group molecular Age** |
| --- | --- | --- |
| **Crown-group ants** | 92 (New Jersey) | 112^42^ |
| **New World army ants** | 16 (Mexican/Dominican) | 27.5^45^ |
| *Eciton* |  | 4.9^45^ |
| *Labidus* |  | 6.91^45^ |
| *Neivamyrmex* | 16 (Mexican/Dominican) | 13.37^45^ |
| *Nomamyrmex* |  | 7.92^45^ |
| **Myrmicinae** | 52.2 (Fushun) | 61^42^ |
| *Acromyrmex* |  | 9^61^ |
| *Aphaenogaster* | 45 (Baltic) |  |
| *Atta* |  | 9^61^ |
| *Crematogaster* | 46 (Kishenehn) | 34.7^60^ |
| *Messor* | 34 (Florissant) | 7.5^60^ |
| *Pheidole* | 45 (Baltic) | 35.2^60^ |
| *Solenopsis* | 35.6 (Kleinkems) | 39.1^60^ |
| *Tetramorium* | 45 (Baltic) | 20.1^60^ |
| **Formicinae** | 92 (New Jersey) | 60^42^ |
| *Lepisiota* |  | 42.9^59^ |
| *Formica* | 44.6 (Bagshot Beds) | 28.6^59^ |
| *Lasius* | 46 (Kishenehn) | 29^59^ |
| **Dolichoderinae** | 78 (Medicine Hat) | 55^42^ |
| *Tapinoma* | 35.6 (Rovno) | 28^62^ |
| **Ponerinae** | 53.5 (Oise) | 73^42^ |
| *Pachycondyla* | 47 (Messel) | 18^63^ |
